# Clinical and toxicological trends in mad honey research: A global bibliometric analysis of scientific evolution (1954–2025)

**DOI:** 10.1097/MD.0000000000050654

**Published:** 2026-09-25

**Authors:** Abdul Samet Sahin, Emre Dilaver, Abdulkadir Gunduz

**Affiliations:** a Department of Emergency Medicine, Karadeniz Technical University, School of Medicine, Trabzon, Turkey; b Department of Emergency Medicine, Kanuni Training and Research Hospital, Trabzon, Turkey.

**Keywords:** bradycardia, grayanotoxin, hypotension, intoxication, mad honey, rhododendron

## Abstract

**Background:**

Honey derived from the nectar of Rhododendron species, known as “mad honey,” possesses unique pharmacological and toxicological properties due to its grayanotoxin content. Prior investigations have largely comprised case reports and experimental studies, lacking a comprehensive overview of broader scientific trends. The present study addresses this gap by providing the first bibliometric analysis of the historical development, thematic directions, and scientific structure of the “mad honey” literature published between 1954 and 2025.

**Methods:**

Data for this retrospective bibliometric analysis were collected from the Web of Science Core Collection database in January 2026. The search strategy used the keywords “mad honey,” “grayanotoxin,” “andromedotoxin,” “rhodotoxin,” and “asebotoxin,” encompassing publications from 1954 to 2025, with no language restrictions.

**Results:**

During the study period, 388 publications were identified across 214 journals, reflecting an annual growth rate of 1.65%. A total of 1253 authors contributed, with an average of 18.57 citations per publication. Country-level analysis revealed that Türkiye leads globally in both publication volume and citation impact, followed by the United States, China, and Japan.

**Conclusions:**

This analysis demonstrates that the mad honey literature has evolved from local case reports toward global clinical research and potential therapeutic applications. Research on mad honey has expanded globally since 2010, with Türkiye leading clinical data and diagnostic protocols. Future studies should shift focus from poisoning cases toward therapeutic potential, product standardization, and safe dosage parameters.

## 1. Introduction

Honey is a natural product produced by honeybees through the biochemical transformation of plant nectar and has been utilized for nutritional and therapeutic purposes in medical practice.^[1,2]^ The botanical origin and environmental conditions of honey are key determinants of its chemical composition and biological characteristics.^[3]^ Accordingly, specific honey types are highlighted in the scientific literature not only for their nutritional value but also for distinctive chemical constituents that confer pharmacological and toxicological properties.

One specific type of honey, referred to as “mad honey” in Türkiye, is derived from the nectar of Rhododendron species that grow in particular geographic regions, predominantly in the Black Sea region.^[4,5]^ The distinctive properties of this honey are due to the presence of grayanotoxin compounds.^[5]^ These diterpenoid compounds prevent sodium channel inactivation, prolonging membrane depolarization and producing significant biological effects, particularly in the cardiovascular and neurological systems.^[6,7]^ Historically, these toxic components were extensively studied under the name andromedotoxin. Descriptions of the toxicity of various Rhododendron species – including their leaves, flowers, and honey – date back to ancient records.^[8]^ This characteristic distinguishes mad honey from other honey types and has established it as a unique subject of scientific research.

The presence of mad honey in human history dates back to antiquity, with historical sources documenting that honey consumed along the Black Sea coast caused transient loss of consciousness and physical weakness among soldiers.^[8]^ These historical records indicate that the effects of mad honey were recognized long before the emergence of modern toxicological knowledge.^[9]^ Currently, mad honey is still used in some communities under the belief that low-dose consumption may confer benefits, and it is employed as a remedy for gastrointestinal disorders, metabolic disturbances, and circulatory system complaints.^[4,10]^ As a result, a significant portion of consumers tends to obtain this product directly from local producers without undergoing standard industrial processing or regulatory inspection.^[11]^ However, its use is limited by a narrow therapeutic window, necessitating careful scientific evaluation.

In the current scientific literature, mad honey is regarded as a multidisciplinary research topic situated at the intersection of botany, pharmacology, toxicology, and clinical medicine.^[7,12,13]^ Accordingly, studies on mad honey cover a wide range of topics, from the biochemical mechanisms of grayanotoxins and experimental model outcomes to clinical findings and public health considerations. However, existing research has predominantly consisted of case reports, review articles, and experimental studies, while comprehensive and synthesizing analyses that quantitatively characterize the broader scientific trends of the field are scarce.

In recent years, bibliometric analyses of honey, bee products, and apitherapy have provided detailed assessments of global research trends, prominent themes, and collaborative networks in honey-related studies.^[2,14-16]^ However, these studies generally address honey as a broad category and do not recognize mad honey as a distinct research domain. Therefore, the present study represents the first bibliometric analysis of mad honey research, with the aim of delineating the historical evolution, thematic orientations, and scientific structure of the literature published between 1954 and 2025.

## 2. Materials and methods

### 2.1. Study design and data source

This study is a retrospective bibliometric analysis providing a comprehensive evaluation of the global literature on “mad honey” and “grayanotoxin.” The research data were retrieved from the Web of Science (WoS) Core Collection database on January 19, 2026, due to its high data quality and comprehensive coverage for academic literature searches.

### 2.2. Search strategy and inclusion criteria

The data collection strategy employed the keywords “mad honey” OR “grayanotoxin” OR “andromedotoxin” OR “rhodotoxin” OR “asebotoxin,” with the “Topic” criterion selected in the WoS database. To maximize the analysis scope, no temporal or language restrictions were applied, and all publications from 1954 to 2025 were included.

Only publications classified as “Article” and “Review” were included in the analysis, while letters, meeting abstracts, book chapters, corrections, and editorial materials were excluded to maintain a focus on high-quality, peer-reviewed, full-text research. The document type was limited to academic journals, and duplicates and irrelevant literature were screened out. Two authors independently reviewed all literature for relevance, achieving 98% agreement; discrepancies were resolved through discussion with a third author.

### 2.3. Data analysis and visualization

The bibliographic data obtained from the WoS database (including authors, titles, publication years, citation counts, and institutional affiliations) were exported in “Plain Text File” format. Two different bibliometric software tools were used for data analysis and scientific mapping. Biblioshiny, the web interface for the R-based Bibliometrix package, was used to compute core bibliometric indicators, including annual scientific output, journal impact factors, h-index values, Bradford’s Law analysis, and citation bursts.^[17]^ The other tool employed was VOSviewer, which was used to visualize keyword co-occurrence networks, co-authorship maps, and international collaboration networks.^[18]^ This tool was preferred for generating network maps that facilitate a clearer understanding of conceptual clusters and the intellectual structure of the literature.

### 2.4. Ethical considerations

Ethical approval was not required for this study, as it was based exclusively on publicly available bibliographic records retrieved from the WoS Core Collection and did not involve human participants, animal subjects, or identifiable personal data.

## 3. Results

### 3.1. Main bibliometric indicators

This bibliometric analysis covers the literature on mad honey published between 1954 and 2025. The scientific exploration of mad honey dates back to early records, with the earliest identified publication in this dataset dating to 1954. Historically, early research was primarily concentrated on experimental pharmacology and characterizing the toxic components, then known as andromedotoxin. The systematic search and selection process resulted in a final dataset of 388 publications, as detailed in the flow diagram (Fig. 1). Of the 388 publications, 29 were identified as review articles. During the study period, 388 publications were identified in 214 distinct scientific journals, with an annual publication growth rate of 1.65%. The main bibliometric indicators are presented in Figure 2.

**Figure 1.**
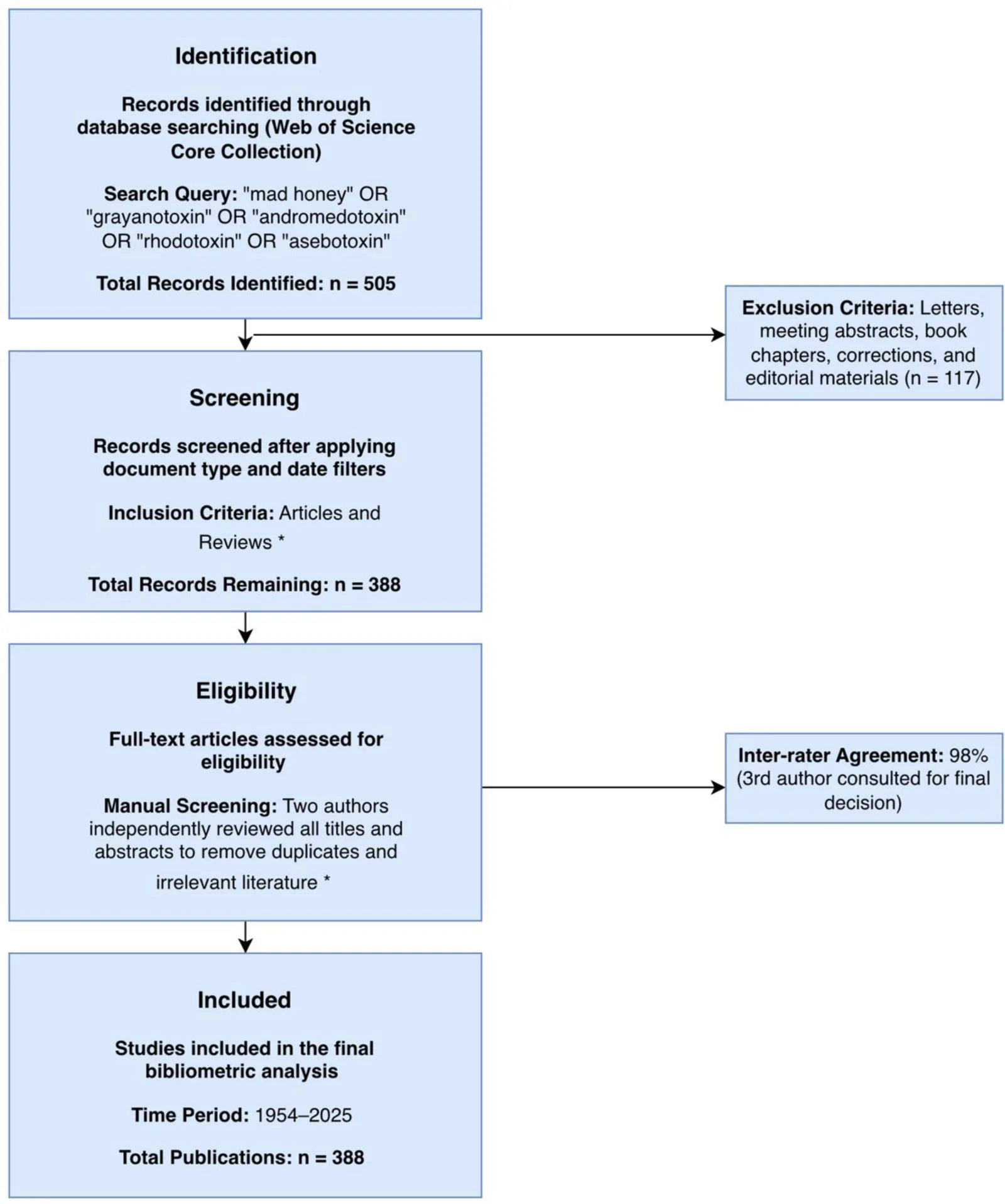
Flowchart of the literature search and study selection process.

**Figure 2.**
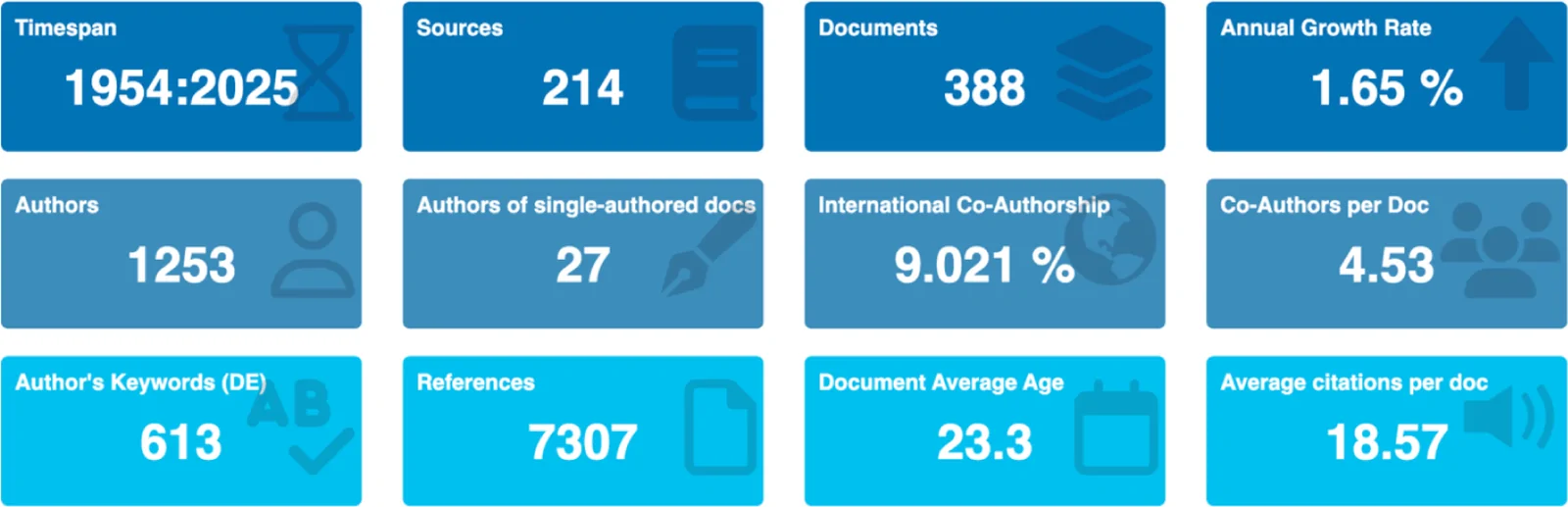
Main bibliometric indicators of the scientific literature (1954–2025).

### 3.2. Annual scientific production and average citation analysis

In the mad honey literature, both annual scientific production and the average number of citations per publication have exhibited notable fluctuations over time. Analysis of the literature from 1954 to 2025 reveals a relatively low level of academic production in the initial period. Annual scientific production and average citation analysis are illustrated in Figure 3.

**Figure 3.**
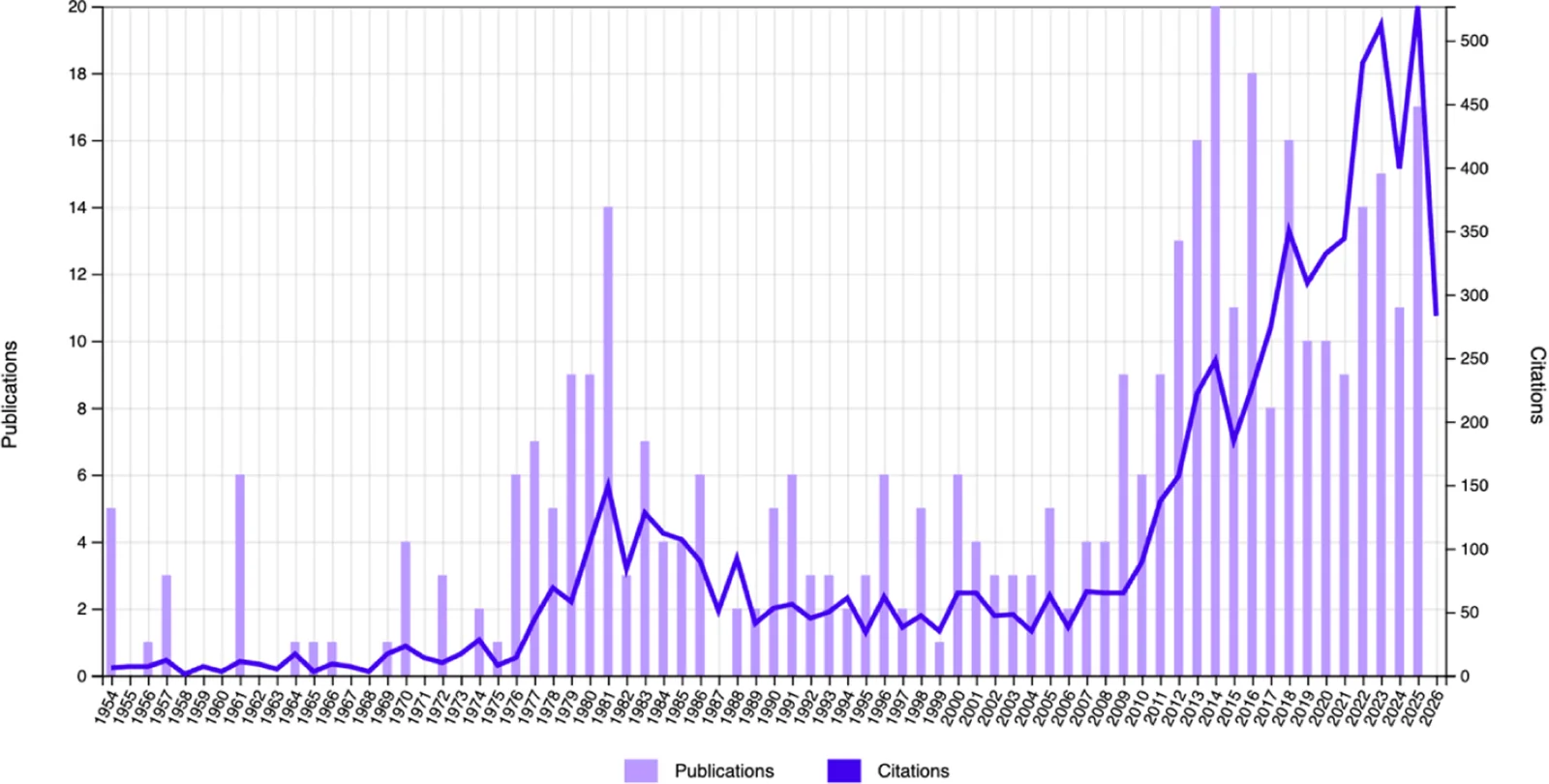
Annual scientific production and citation trends (1954–2025).

### 3.3. Journal (source) analysis

The mad honey literature demonstrates a concentration of publications within a limited number of journals. During the analyzed period, the journal with the most publications was the Journal of Pharmacology and Experimental Therapeutics, which published 17 articles. This was followed by Agricultural and Biological Chemistry and Tetrahedron Letters, each with 9 publications. The journals receiving the highest number of local citations and the local impact of journals are presented in Table 1.

**Table 1:** Performance and impact analysis of leading journals based on bibliometric indicators.

Journal	h_index	g_index	m_index	TC	NP	PY_start
Journal of Pharmacology And Experimental Therapeutics	13	17	0.17808219	498	17	1954
Phytochemistry	7	8	0.14583333	234	8	1979
Chemical and Pharmaceutical Bulletin	6	7	0.125	89	7	1979
Journal of Natural Products	6	8	0.125	233	8	1979
Journal of the American Chemical Society	6	7	0.08219178	194	7	1954
Tetrahedron Letters	6	9	0.09090909	204	9	1961
Agricultural and Biological Chemistry	5	9	0.07575758	86	9	1961
Journal of Agricultural and Food Chemistry	5	5	0.18518519	111	5	2000
Yakugaku Zasshi-Journal of the Pharmaceutical Society of Japan	5	5	0.09433962	53	5	1974
Bulletin of the Chemical Society of Japan	4	4	0.08510638	31	4	1980
Food and Chemical Toxicology	4	5	0.2	167	5	2007
Journal of Ethnopharmacology	4	7	0.11764706	254	7	1993
Turkish Journal of Emergency Medicine	4	5	0.21052632	59	5	2008
Biochemical and Biophysical Research Communications	3	3	0.06	76	3	1977
Clinical Toxicology	3	3	0.15789474	152	3	2008

NP = number of publications, PY_start = publication year start, TC = total citations.

### 3.4. Institutional analysis

#### 3.4.1. Most relevant affiliations

Institutional analysis revealed that Karadeniz Technical University was the most productive institution in mad honey research, with 55 published articles. Following Duzce University (31 publications), Hiroshima University (26 publications), Erciyes University (23 publications), and Chinese Academy of Sciences (15 publications) were also identified as major contributors to the mad honey literature. The most productive institutions are shown in Figure 4.

**Figure 4.**
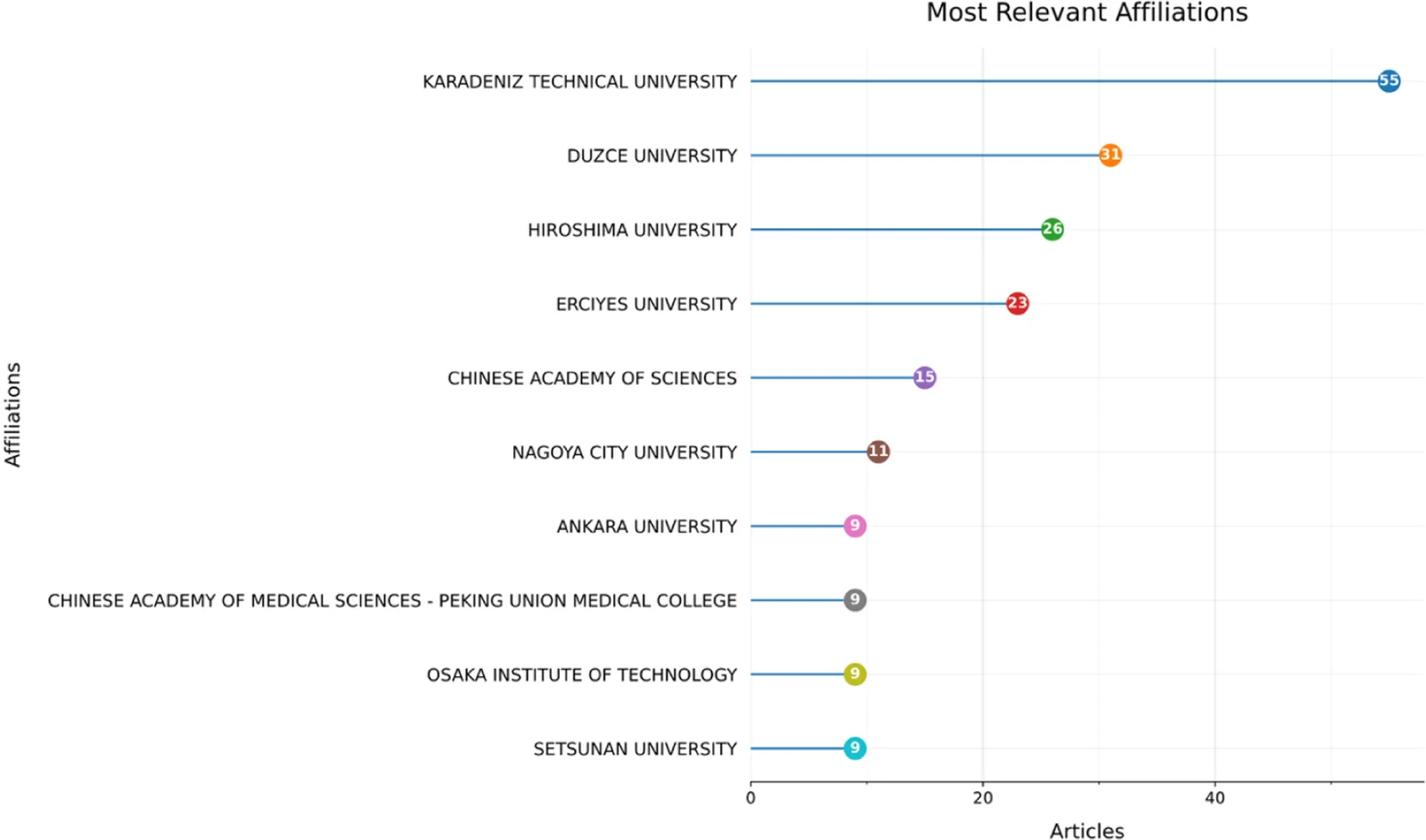
Most relevant affiliations by number of articles (1954–2025).

#### 3.4.2. Institutional publication performance over time

When institutional publication trends over time are examined, it becomes evident that contributions were initially limited to a small number of institutions, followed by a pronounced rise in institutional output after the 2000s. A consistent growth in publication output was observed for Karadeniz Technical University over time, whereas Duzce University and Erciyes University exhibited a pronounced acceleration in publication activity in recent years. The contributions of institutions based in China and Japan were found to be concentrated in later periods.

### 3.5. Country analysis and citation dynamics

#### 3.5.1. Scientific production by country

Analysis of country-level scientific output reveals that research on mad honey is geographically concentrated in certain regions. Map-based analyses reveal that the countries with the highest publication output are Türkiye, Japan, the United States, and China. In addition, it was determined that Nepal, several European countries, and Australia have also contributed to the literature. Overall, the distribution indicates that publication output is predominantly centered in Asia and Europe. Scientific production by country and countries’ publication output over time are presented in Figures 5 and 6. The distribution of publication performance and international collaboration rates by country is presented in Table 2.

**Table 2:** Distribution of publication performance and international collaboration rates by country.

Country	Articles	Articles %	SCP	MCP	MCP %
Turkey	93	23.907455	88	5	5.37634409
Japan	50	12.8534704	50	0	0
United States	46	11.8251928	42	4	8.69565217
China	44	11.311054	38	6	13.6363636
Nepal	15	3.85604113	13	2	13.3333333
Germany	13	3.34190231	11	2	15.3846154
Korea	11	2.8277635	9	2	18.1818182
United Kingdom	6	1.54241645	5	1	16.6666667
France	4	1.02827763	4	0	0
Poland	4	1.02827763	4	0	0
Austria	3	0.77120823	3	0	0
Ireland	3	0.77120823	0	3	100
Netherlands	3	0.77120823	1	2	66.6666667
Australia	2	0.51413882	1	1	50
Brazil	2	0.51413882	2	0	0

MCP = multiple country publication, SCP = single country publication.

**Figure 5.**
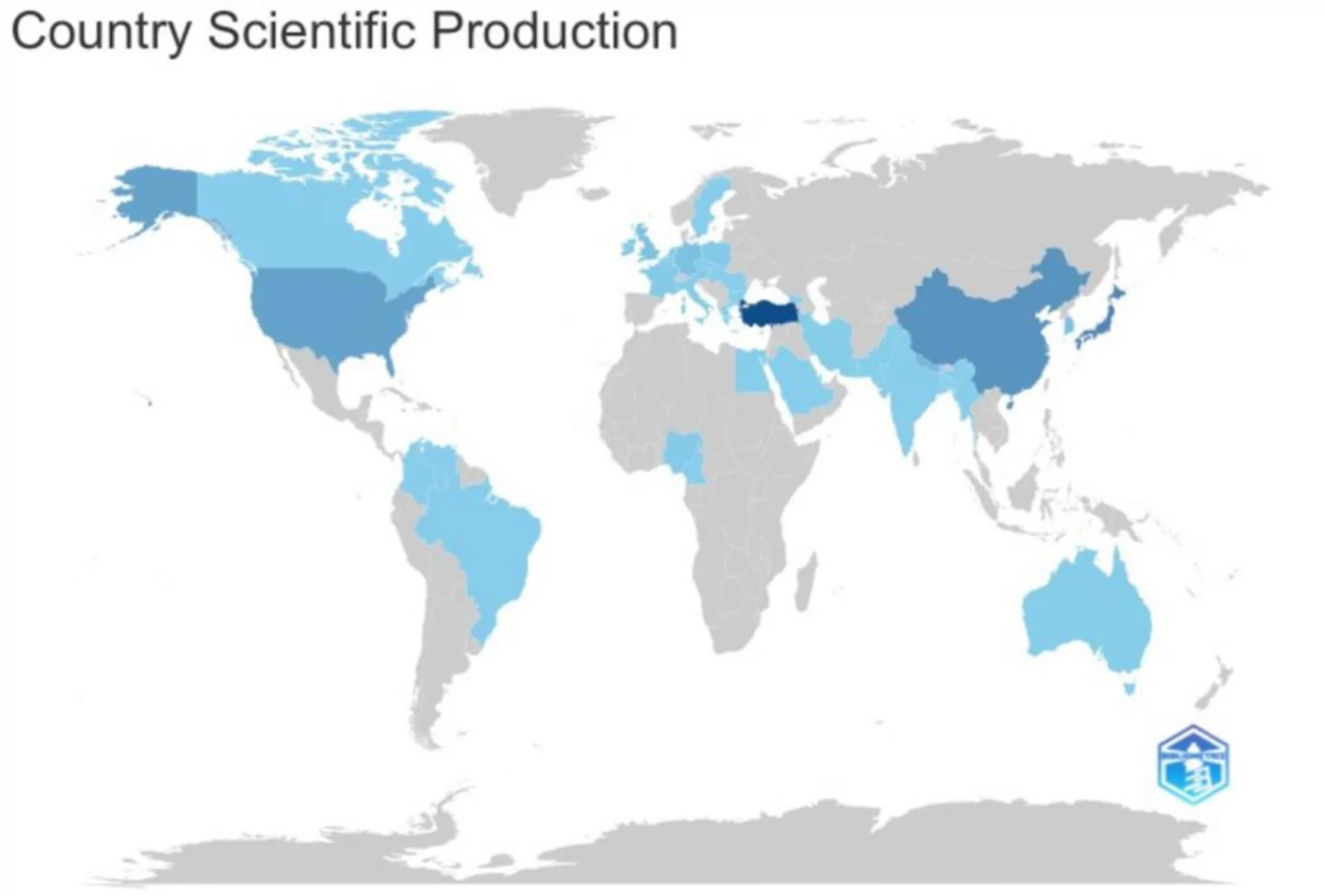
Geographical distribution of scientific production by country (1954–2025).

**Figure 6.**
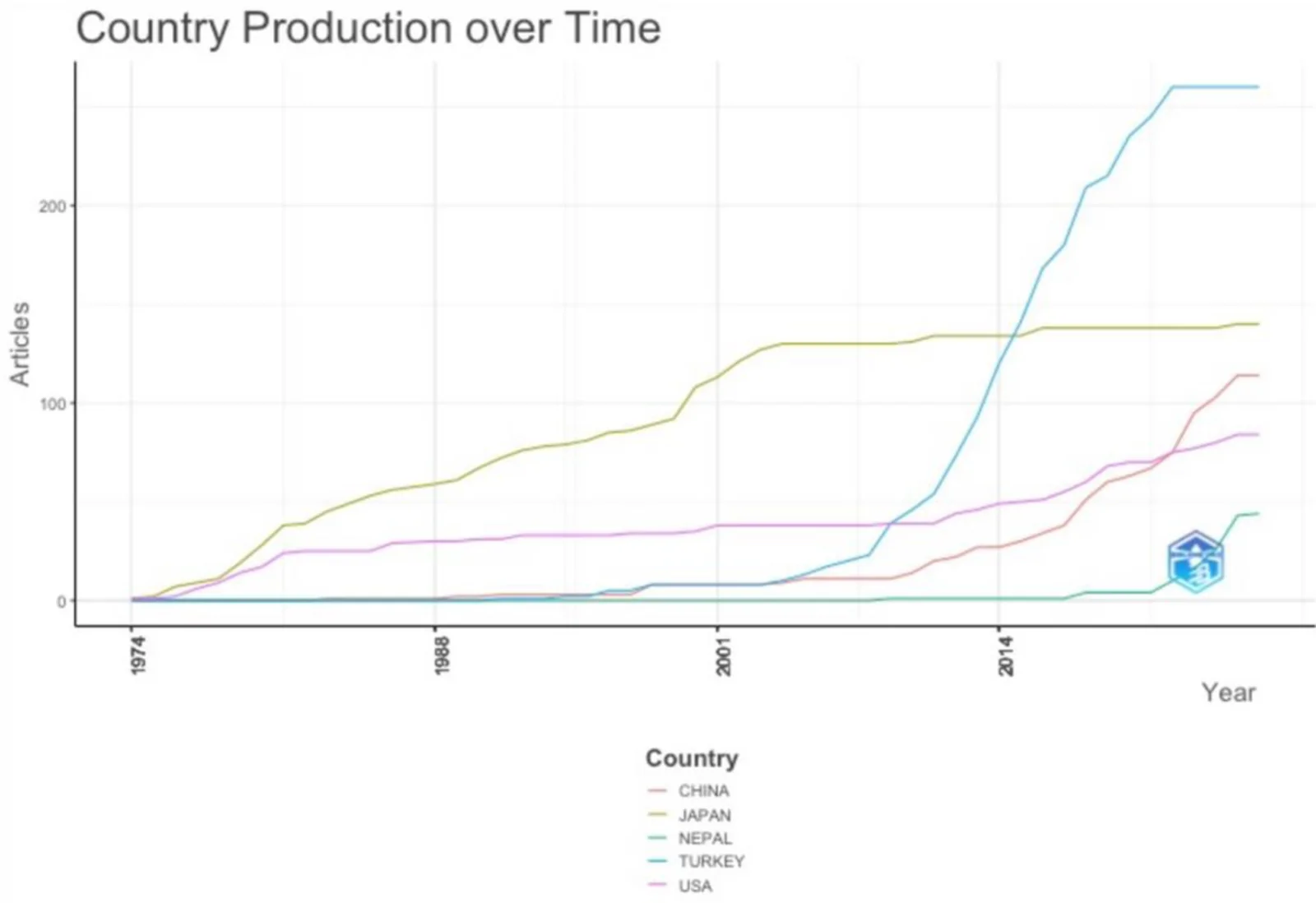
Growth of scientific articles by country over time.

#### 3.5.2. Most cited countries

Citation analysis demonstrates that Türkiye leads the mad honey literature with over 1460 citations, followed by the United States. China ranks third, with approximately 1006 citations. Japan, Austria, South Korea, Ireland, France, Poland, and the Netherlands follow these leading countries. This distribution suggests that, beyond publication volume, specific countries exert substantial citation impact within the literature. The distribution of citation counts by country is presented in Figure 7.

**Figure 7.**
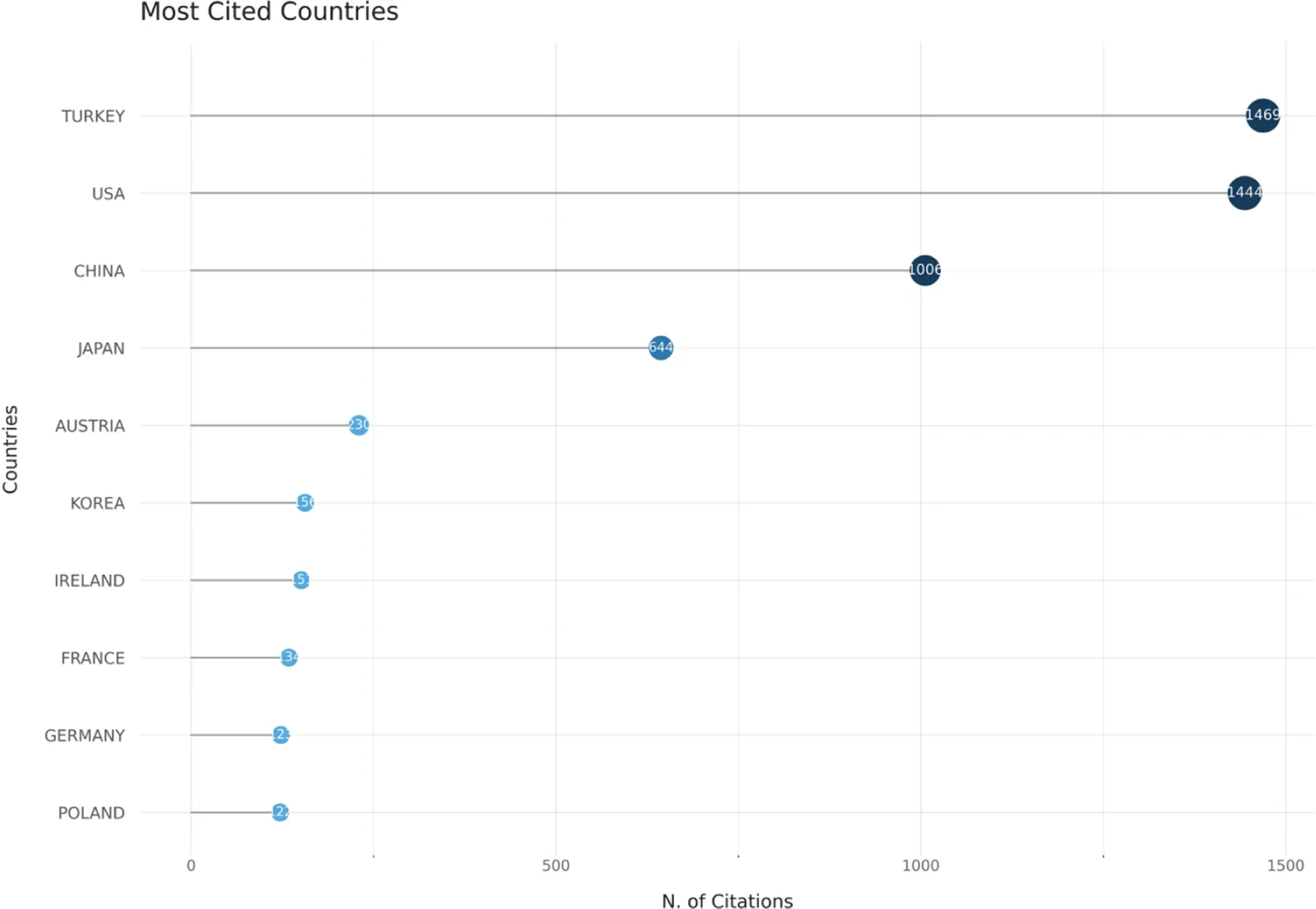
Citation analysis of the top countries in the mad honey literature.

#### 3.5.3. Reference publication year spectroscopy (RPYS)

Reference publication year spectroscopy (RPYS) analysis indicates that the references underpinning the mad honey literature are concentrated in specific time periods. Examination of the distribution of cited references by publication year shows that citation counts remained at very low levels in the period prior to 1950. From the 1960s onward, the total number of cited references (represented by the black line) increased, reaching approximately 150 to 200 during the period between 1970 and 1980. After the early 2000s, the number of cited references increased markedly, exceeding 400 during 2008 to 2010. In subsequent years, a declining trend in the number of cited references was observed, with values ranging from approximately 120 to 150 in the post-2020 period. The 5-year median deviation values, represented by the red line, fluctuated over time, taking on positive values in some years and negative values in others. RPYS is presented in Figure 8.

**Figure 8.**
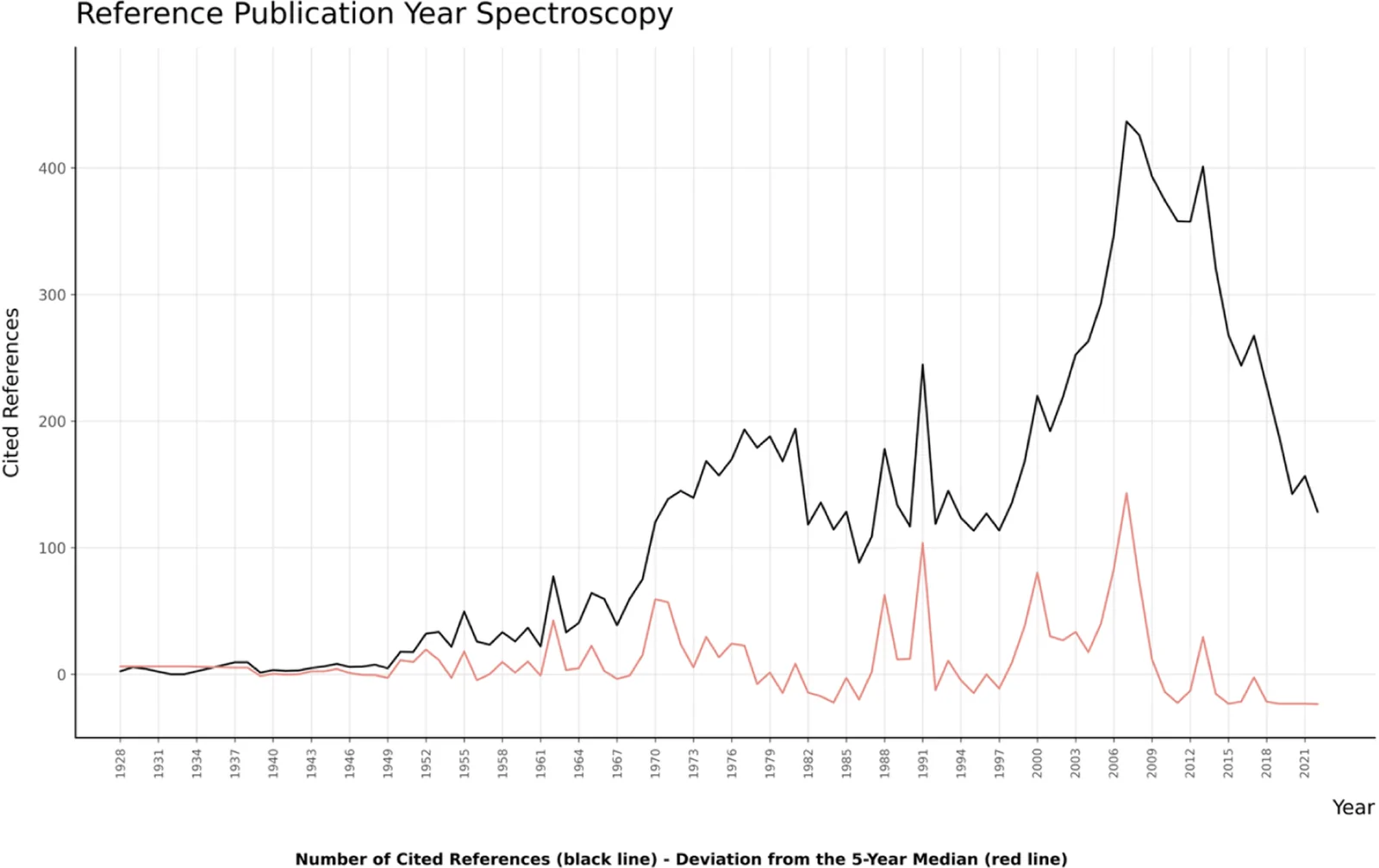
Reference publication year spectroscopy (RPYS) of the mad honey literature. RPYS = reference publication year spectroscopy.

### 3.6. Keyword analysis

#### 3.6.1. Keyword distribution, frequency and trend topics

Keyword frequency analysis demonstrates that certain concepts are central within the mad honey literature. The most frequently used keyword was “mad honey,” appearing 116 times (13% of all keywords), followed by “Na” (121 occurrences; 13%) and “grayanotoxin” (91 occurrences; 10%). Additional high-frequency keywords included “bradycardia” (43; 5%), “intoxication” (33; 4%), “rhododendron” (33; 4%), “honey” (30; 3%), “grayanotoxins” (25; 3%), and “hypotension” (25; 3%). When examining the chronological flow, it is seen that fundamental physiological terms such as “mechanism” (1996), “skeletal-muscle” (1999), and “batrachotoxin” (2000) were dominant in the late 1990s. At the beginning of the 2000s, studies at the molecular level such as “channels” (2002), “d-ring” (2002), and “point-mutations” (2002) came to the fore, while terms such as “Turkey” and “grayanotoxin-i” (2009) entered the literature as of 2008. During the period between 2010 and 2020, terms related to clinical symptoms such as “poisoning” (2014), “syncope” (2014), “bradycardia” (2016), “intoxication” (2016), and “hypotension” (2016). Looking at the current period of the data (2020–2025), it is observed that the focus has shifted to biochemical and taxonomic terms such as “antioxidant” (2020), “diterpenoids” (2022), “biological-activities” (2023), and “ericaceae family” (2023). In the most recent data from 2024 and 2025, the terms “identification,” “nepal,” and “pollen” have entered the system as the median year. Figures 9 and 10 present the keyword distribution and frequency.

**Figure 9.**
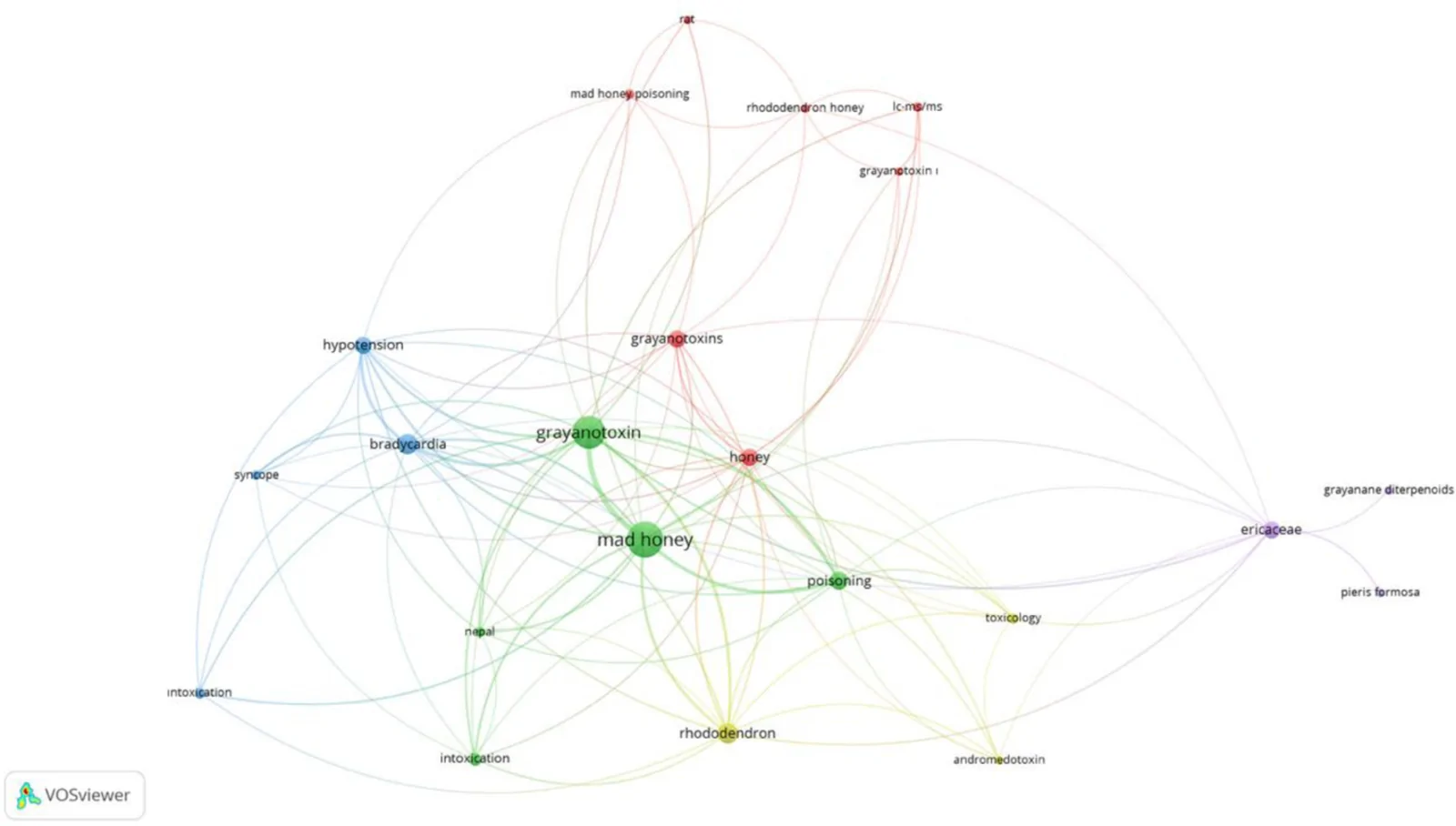
Word cloud analysis of the keywords in mad honey literature.

**Figure 10.**
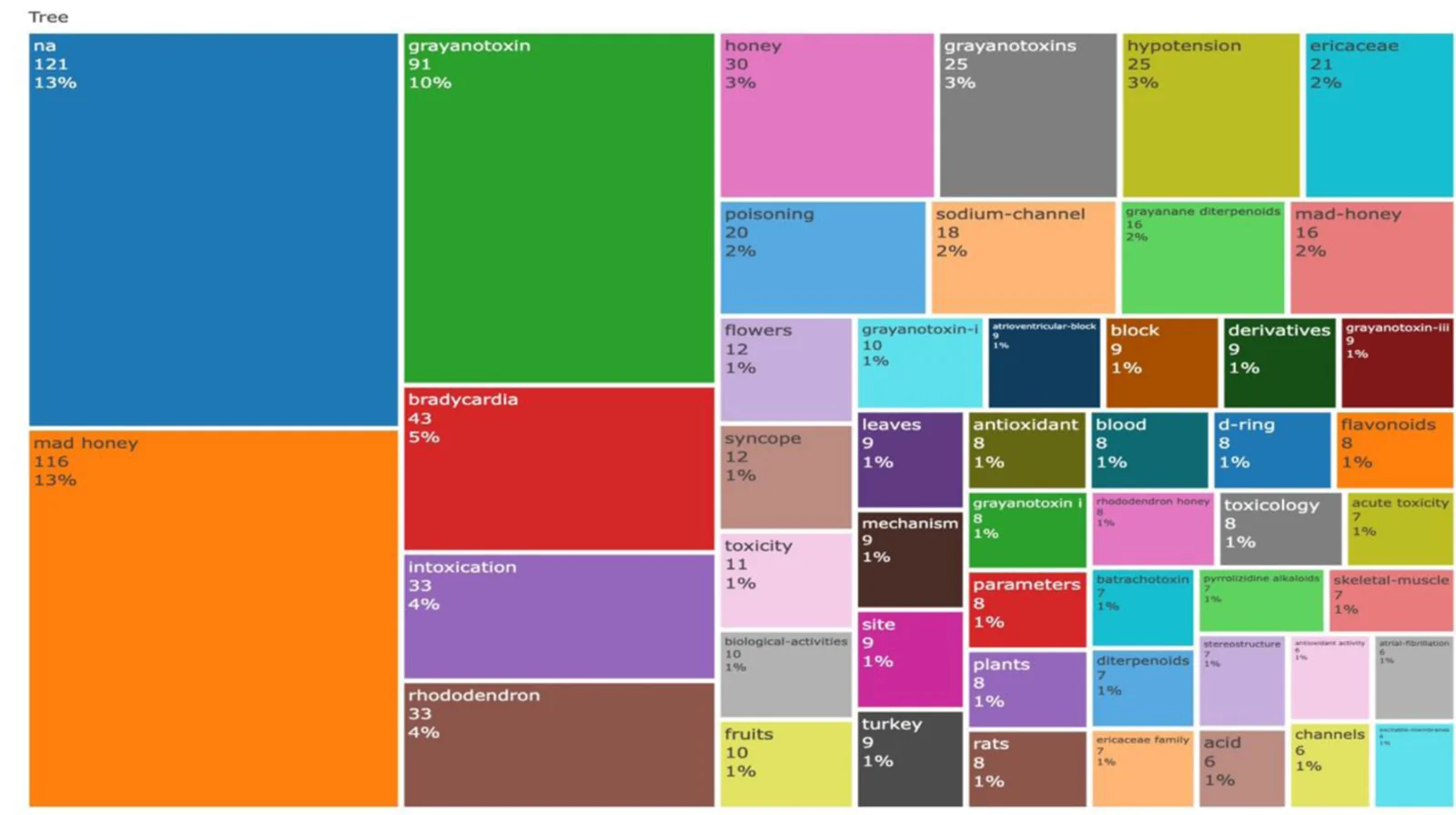
Treemap analysis of keyword distribution and frequency.

#### 3.6.2. Keyword co-occurrence network

Keyword co-occurrence network analysis indicates that “mad honey” and “grayanotoxin” are central concepts in the literature. These terms are closely linked with clinical and botanical keywords such as “intoxication,” “bradycardia,” “hypotension,” and “rhododendron.” The network structure reveals several distinct clusters.

### 3.7. Article relationship analysis

Article relationship analysis reveals the structural relationships among studies in the mad honey literature based on shared references. The results of the network analysis show that the literature is organized into several interrelated clusters instead of a uniform structure. The article relationship analysis is presented in Figure 11.

**Figure 11.**
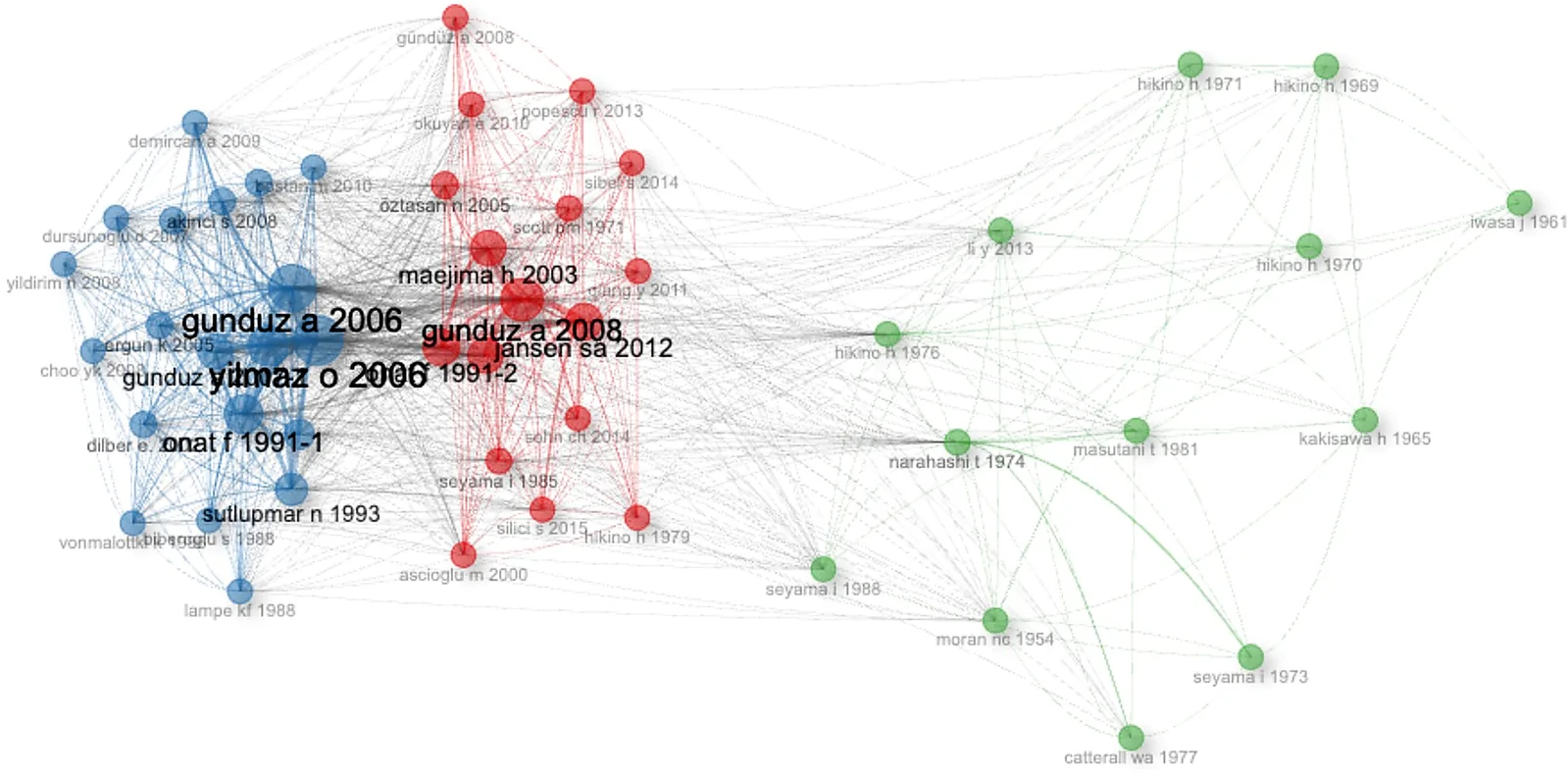
Article relationship analysis of the mad honey literature based on shared references.

Three distinct main clusters were identified within the network structure. The first cluster is located at the core of the literature and primarily comprises clinical research conducted by Gunduz et al Studies within this cluster predominantly consist of articles published between 2006 and 2008, focusing on clinical findings, emergency department presentations, and cardiac effects. The second cluster predominantly comprises earlier studies and is structured around publications by Yilmaz (2006), Onat (1991), and related works. This cluster encompasses studies concentrating on the pharmacological actions and experimental mechanisms of grayanotoxins, serving as seminal works that laid the groundwork for later clinical investigations. The third cluster mainly comprises early experimental and pharmacological studies published between 1960 and 1980.

### 3.8. Intercountry scientific collaboration analysis

An analysis of international scientific collaboration reveals the geographic distribution of international co-authorship relationships within the mad honey literature. Map-based network analysis demonstrates that, in addition to publication output, scientific collaborations are also concentrated around specific countries.

According to the analysis, Türkiye occupies a central position in the international collaboration network and exhibits direct connections with numerous countries. In particular, the collaborative links between Türkiye and the United States, Japan, and China are more pronounced. Collaboration links among these countries are represented by thicker and denser connections within the network.

## 4. Discussion

The bibliometric analysis of the “mad honey” literature covering the period from 1954 to 2025 demonstrates a clear increase in publication trends over time. The increasing trend in publication numbers, particularly in recent years, parallels the overall growth of scientific output in the literature. A similar pattern has been reported in other areas related to honey and health; for instance, studies on the antimicrobial and antioxidant properties of honey were limited in the early 2000s but accelerated markedly after the 2010s, exhibiting an exponential annual growth rate of approximately 17.5%.^[14]^ Likewise, the global literature on honey and diabetes has shown a significant increasing trend during the last 10 years.^[19]^ Similarly, publication activity in the field of mad honey remained limited during the mid-20th century but has accelerated since the 2000s, gaining notable momentum in recent years. This finding indicates that the topic has attracted increasing attention within the scientific community and has evolved in alignment with broader research trends.

Bibliometric data suggest that author collaboration networks in mad honey research are relatively limited in size but remain focused and specialized. When compared with similar bibliometric analyses, it is expected that the proportion of international collaborative studies remains relatively low in a niche field such as mad honey research. Indeed, global research on the health effects of honey has been reported to involve an average of approximately 5 authors per article, with nearly 27% of studies conducted through international collaboration.^[14]^ This proportion may be more limited in the context of mad honey, as the topic is investigated by a geographically and scientifically specific research community. On the other hand, examination of the relevant literature highlights strong domestic collaborations and network structures centered around specific countries. For example, in bee venom research, South Korea has been reported as one of the main hubs, establishing dense collaborative links both among its domestic institutions and at the international level.^[15]^ Similarly, in mad honey research, researchers in Türkiye appear to engage in dense domestic collaborations, while international networks remain relatively limited. Accordingly, the mad honey literature differs from honey and bee product research that has established extensive global collaboration networks; however, it reflects a collaboration dynamic similar to that observed in topics requiring region-specific expertise.

Country-based analysis indicates that mad honey research output is predominantly concentrated in Türkiye. This is an expected finding, as the mad honey phenomenon is historically and botanically associated with limited geographic areas, particularly the Black Sea region of Türkiye.^[7]^ As noted by Santos-Buelga and González-Paramás, although Rhododendron species are distributed globally from Japan to the United States, the majority of poisoning cases continue to be reported from Turkey. This regional concentration is attributed not only to the local prevalence of toxic species like *R ponticum* and *R luteum* but also to specific beekeeping practices and local consumption habits.^[20]^ For example, in global research on the medicinal properties of honey, numerous countries, including India, Italy, the United States, and Türkiye, have been identified among the most productive, each contributing approximately 25 to 30 publications.^[14]^ Likewise, bibliometric evaluations of propolis research have shown that Brazil, China, and Türkiye collectively dominate the literature, reflecting a more balanced international distribution of research output.^[16]^ In the context of mad honey, the predominance of Türkiye can be attributed to the regional specificity of the phenomenon, whereas in other bee products or apitherapy-related topics, scientific leadership appears to be shared among different countries. A similar comparison can also be made at the institutional level: The most productive institutions in the mad honey literature are predominantly universities in Türkiye, particularly Karadeniz Technical University, which also ranks among the leading contributors to the broader international honey research landscape.^[14]^ This finding indicates that research on mad honey is largely concentrated in local centers of expertise, while these centers simultaneously constitute integral components of the global honey research network. Beyond publication counts, this concentration carries strategic implications. Indeed, bibliometric network analysis reveals that, despite Türkiye’s quantitative and citation-based dominance, the field exhibits a relatively inward-looking collaboration structure, with a multiple country publication (MCP) rate of only 5.38%. Whereas the international co-authorship rate in the broader apitherapy literature is approximately 27%,^[14]^ in the mad honey literature it remains around 9%. Japan (12.85% of publications; 0% MCP) is oriented almost entirely toward domestic research, whereas the United States (11.83%; 8.70% MCP) and China (11.31%; 13.64% MCP) display markedly higher international integration. This geographic and methodological divergence carries an important secondary implication: researchers in endemic regions such as Türkiye and Nepal predominantly generate clinical emergency medicine, case-series, and epidemiological data, whereas centers in the United States, Japan, and China specialize in advanced organic synthesis, D-ring modifications, ion-channel electrophysiology, and metabolomics. To bridge this persistent gap between clinical field data and advanced molecular research, integrating the clinical data accumulated in endemic regions with global analytical and pharmacological laboratories through formal consortia represents a strategic necessity.

Content clustering based on keyword analysis demonstrated that the mad honey literature is divided into clearly defined subthemes. The findings suggest that publications are predominantly clustered around themes such as intoxication cases and toxicological effects, the cardiovascular consequences of grayanotoxin, and the potential medical applications of mad honey. This structure is consistent with patterns observed in other topics examined using similar bibliometric approaches. For example, bibliometric mapping of bee venom research has demonstrated that studies cluster around 3 main themes: the components of bee venom, allergic and immunological responses, and clinical therapeutic applications.^[15]^ Similarly, publications on honey and diabetes have been reported to show keyword concentrations around clinical outcome–oriented themes such as human studies, glycemic control, and wound healing.^[19]^ Furthermore, the recent European Food Safety Authority (EFSA) scientific opinion introduced a protective threshold of 0.05 mg/kg for grayanotoxins I and III in honey. This regulatory development highlights a shift in the literature from simple case reporting toward food safety and toxicological risk assessment. The fact that Turkey ranks first in citation impact suggests that Turkish researchers are providing the essential clinical and toxicological data required for such international safety standards.^[21]^ In line with these observations, mad honey studies similarly form internal subclusters that shape the overall structure of the literature. In particular, the toxicological and public health dimension and the pharmacological potential dimension emerge as 2 prominent focal areas in the keyword analysis. This indicates that, although mad honey research represents a relatively limited field, it has matured into distinct subdisciplines and exhibits a developmental trajectory consistent with findings from other bibliometric analyses.

## 5. Strengths and limitations

The strengths of this study include its status as the first bibliometric synthesis dedicated specifically to mad honey, its coverage of more than 7 decades without language restriction, and its use of 2 complementary tools (Bibliometrix/Biblioshiny and VOSviewer) for methodological triangulation. Nonetheless, several limitations should be acknowledged. This analysis includes only journals indexed in the WoS database. Publications written in local languages, indexed exclusively in other databases, or not included in international indexing systems (e.g., studies published only in national databases in Turkish or Japanese) may therefore have been excluded from the analysis. Finally, as the search relied on predefined keywords and automated bibliometric extraction, records indexed under atypical terminology may have been missed, and citation-based indicators inevitably favor older publications that have had more time to accumulate citations.

## 6. Conclusion

This study provides the first bibliometric analysis of the “mad honey” literature from 1954 to 2025, demonstrating a significant acceleration in scientific output, especially after 2010. Türkiye, due to its geographic context and the contributions of local universities, occupies a leading position globally in both publication volume and citation impact. While existing research has primarily addressed intoxication cases and toxicological effects such as bradycardia and hypotension, further exploration of the therapeutic properties of grayanotoxins is essential for advancing the field. Future research should prioritize product standardization, determination of safe therapeutic dose ranges, assessment of pharmacological applicability, and broader international collaboration to translate region-specific clinical expertise into globally applicable evidence.

## Author contributions

**Conceptualization:** Abdul Samet Sahin, Emre Dilaver, Abdulkadir Gunduz.

**Data curation:** Abdul Samet Sahin, Emre Dilaver, Abdulkadir Gunduz.

**Formal analysis:** Abdul Samet Sahin, Abdulkadir Gunduz.

**Investigation:** Abdul Samet Sahin, Emre Dilaver, Abdulkadir Gunduz.

**Methodology:** Abdul Samet Sahin, Abdulkadir Gunduz.

**Project administration:** Abdul Samet Sahin.

**Resources:** Emre Dilaver.

**Software:** Abdul Samet Sahin.

**Supervision:** Abdul Samet Sahin, Emre Dilaver, Abdulkadir Gunduz.

**Validation:** Emre Dilaver.

**Visualization:** Emre Dilaver, Abdulkadir Gunduz.

**Writing – original draft:** Abdul Samet Sahin, Abdulkadir Gunduz.

**Writing – review & editing:** Abdul Samet Sahin, Emre Dilaver, Abdulkadir Gunduz.

## References

[1] Miguel MG Antunes MD Faleiro ML. Honey as a complementary medicine. Integr Med Insights. 2017;12:1178633717702869. 10.1177/1178633717702869 PMC5406168 28469409

[2] Zakaria R Ahmi A Ahmad AH. Visualising and mapping a decade of literature on honey research: a bibliometric analysis from 2011 to 2020. J Apic Res. 2021;60:359–68.

[3] Ahmed S Sulaiman SA Baig AA. Honey as a potential natural antioxidant medicine: an insight into its molecular mechanisms of action. Oxid Med Cell Longev. 2018;2018:8367846. 10.1155/2018/8367846 PMC5822819 29492183

[4] Koca I Koca AF. Poisoning by mad honey: a brief review. Food Chem Toxicol. 2007;45:1315–8. 10.1016/j.fct.2007.04.006 17540490

[5] Gunduz A Turedi S Russell RM Ayaz FA. Clinical review of grayanotoxin/mad honey poisoning past and present. Clin Toxicol (Phila). 2008;46:437–42. 10.1080/15563650701666306 18568799

[6] Gunduz A Durmus I Turedi S Nuhoglu I Ozturk S. Mad honey poisoning-related asystole. Emerg Med J. 2007;24:592–3. 10.1136/emj.2006.045625 PMC2660097 17652692

[7] Ullah S Khan SU Saleh TA Fahad S. Mad honey: uses, intoxicating/poisoning effects, diagnosis, and treatment. RSC Adv. 2018;8:18635–46. 10.1039/c8ra01924j PMC9080652 35541133

[8] Wood HB Jr Stromberg VL Keresztesy JC. Andromedotoxin. A potent hypotensive agent from rhododendron maximum. J Am Chem Soc. 1954;76:5689–92.

[9] Gunduz A Turedi S Uzun H Topbas M. Mad honey poisoning. Am J Emerg Med. 2006;24:595–8. 10.1016/j.ajem.2006.01.022 16938599

[10] Samarghandian S Farkhondeh T Samini F. Honey and health: a review of recent clinical research. Pharmacognosy Res. 2017;9:121–7. 10.4103/0974-8490.204647 PMC5424551 28539734

[11] Silici S Atayoglu AT. Mad honey intoxication: a systematic review on the 1199 cases. Food Chem Toxicol. 2015;86:282–90. 10.1016/j.fct.2015.10.018 26547022

[12] Aryal M. Grayanotoxins in mad honey: mechanisms of toxicity, clinical management, and therapeutic implications. J Appl Toxicol. 2025;45:2240–56. 10.1002/jat.4855 40635392

[13] Jansen SA Kleerekooper I Hofman ZL Kappen IF Stary-Weinzinger A van der Heyden MA. Grayanotoxin poisoning: ‘mad honey disease’ and beyond. Cardiovasc Toxicol. 2012;12:208–15. 10.1007/s12012-012-9162-2 PMC3404272 22528814

[14] Stefanis C Stavropoulou E Giorgi E. Honey’s antioxidant and antimicrobial properties: a bibliometric study. Antioxidants (Basel). 2023;12:414. 10.3390/antiox12020414 PMC9952334 36829972

[15] Şenel E. A holistic analysis of bee venom literature: bibliometric evaluation of the global publication productivity on bee venom between 1975 and 2017. Hitit Med J. 2019;1:6–11.

[16] Wang Y Liu X Yang Y. Bibliometric analysis of propolis research based on ITGInsight. Rev Bras Farmacogn. 2026;36:22.

[17] Aria M Cuccurullo C. bibliometrix: an R-tool for comprehensive science mapping analysis. J Informetr. 2017;11:959–75.

[18] van Eck NJ Waltman L. Software survey: VOSviewer, a computer program for bibliometric mapping. Scientometrics. 2010;84:523–38. 10.1007/s11192-009-0146-3 PMC2883932 20585380

[19] Bobiş O Dezmirean DS Moise AR. Honey and diabetes: the importance of natural simple sugars in diet for preventing and treating different type of diabetes. Oxid Med Cell Longev. 2018;2018:4757893. 10.1155/2018/4757893 PMC5817209 29507651

[20] Santos-Buelga C González-Paramás AM Chemical composition of honey. In: Alvarez-Suarez JM, ed. Bee Products – Chemical and Biological Properties. 2nd ed. Springer Nature Switzerland AG; 2025:47–104.

[21] EFSA Panel on Contaminants in the Food Chain (CONTAM); Schrenk D Bignami M Bodin L. Scientific Opinion on the risks for human health related to the presence of grayanotoxins in certain honey. EFSA J. 2023;21:e07866. 10.2903/j.efsa.2023.7866 PMC9978999 36875862

